# Increased Oxidative Phosphorylation Is Required for Stemness Maintenance in Liver Cancer Stem Cells from Hepatocellular Carcinoma Cell Line HCCLM3 Cells

**DOI:** 10.3390/ijms21155276

**Published:** 2020-07-25

**Authors:** Ge Liu, Qing Luo, Hong Li, Qiuping Liu, Yang Ju, Guanbin Song

**Affiliations:** 1 Key Laboratory of Biorheological Science & Technology, Ministry of Education, College of Bioengineering, Chongqing University, Chongqing 400030, China; liuge@cqu.edu.cn (G.L.); qing.luo@cqu.edu.cn (Q.L.); li_hong@cqu.edu.cn (H.L.); liuqp@cqu.edu.cn (Q.L.); 2 Department of Mechanical Science and Engineering, Nagoya University, Nagoya 464-8603, Japan; ju@mech.nagoya-u.ac.jp

**Keywords:** hepatocellular carcinoma, cancer stem cells, oxidative phosphorylation, glycolysis, cell metabolism

## Abstract

Cancer stem cells (CSCs) are considered to be the main cause of tumor recurrence, metastasis, and an unfavorable prognosis. Energy metabolism is closely associated with cell stemness. However, how the stemness of liver cancer stem cells (LCSCs) is regulated by metabolic/oxidative stress remains poorly understood. In this study, we compare the metabolic differences between LCSCs and the hepatocellular carcinoma cell line HCCLM3, and explore the relationship between metabolism and LCSC stemness. We found that LCSCs from the hepatocellular carcinoma cell HCCLM3 exhibited more robust glucose metabolism than HCCLM3, including glycolysis, oxidative phosphorylation (OXPHOS), and pyruvate produced by glycolysis entering mitochondria for OXPHOS. Moreover, 2-deoxy-D-glucose (2-DG) enhanced the LCSC stemness by upregulating OXPHOS. In contrast, Mdivi-1 reduced the levels of OXPHOS and weakened the stemness by inhibiting mitochondrial fission. Together, our findings clarify the relationship between energy metabolism and LCSC stemness and may provide theoretical guidance and potential therapeutic approaches for liver cancer.

## 1. Introduction

Liver cancer is a serious threat to human health. In developing countries, the three deadliest cancers are liver cancer, stomach cancer, and cervical cancer [1]. As liver cancer exhibits chemoradiotherapeutic resistance, and most patients are only diagnosed at an advanced stage, the 5-year survival rate for liver cancer patients is only 10.1% [2], imposing a heavy burden on society. One reason for the lack of the efficacy of current therapies may be their inability to effectively target cancer stem cells (CSCs) or tumor-initiating cells (TICs). These cells, residing at the top of tumor heterogeneity, are inherently resistant to chemotherapy and ionizing radiation, leading to treatment resistance and diffusion [3,4].

Energy metabolism is closely associated with cell stemness. Previous studies have revealed that human embryonic stem cells (ESCs), hematopoietic stem cells (HSCs), mesenchymal stem cells (MSCs), neural stem cells (NSCs), and induced pluripotent stem cells (iPSCs) are dependent on glycolysis for their energy supply. Oxidative phosphorylation (OXPHOS) is the energy production pathway active during differentiation, demonstrating that the transformation of energy metabolism is a prerequisite for stem cell differentiation. Similarly, during the formation of iPSCs, the expression of stemness-related genes following metabolic transformation suggests that energy metabolism plays a crucial role in stem cell maintenance [5,6]. However, the relationship between energy metabolism and the stemness of liver cancer stem cells (LCSCs) is incompletely understood. The study of inducing differentiation or reducing stemness by regulating LCSC energy metabolism has recently attracted interest and attention as a strategy for liver cancer therapy.

In 1923, Otto Warburg discovered that proliferating ascite cancer cells can convert most of their glucose into lactic acid, even in the presence of oxygen. This phenomenon of high glucose absorption, aerobic glycolysis, and high lactic acid production is called the Warburg effect [7]. This Warburg effect is beneficial not only for the generation of metabolic intermediates important for tumor macromolecular biosynthesis, but also for cellular bioenergetics. Despite intensive studies documenting that cancer cells rely on glycolysis, glycolytic inhibitors such as 2-deoxy-D-glucose (2-DG) have little or no effect on solid tumor growth in clinical settings [8,9]. The mechanisms for the lack of sensitivity of cancer cells to glycolytic inhibition still need to be characterized. A recent study illustrated that metabolic reprogramming underlies the escape of cancer cells from glycolytic addiction [10]. Currently, little is known about how CSCs respond to glycolytic inhibition. The metabolic reprogramming of CSCs may contribute to the resistance of cancers to glycolysis inhibition and play a critical role in tumor cell survival and tumor progression under inhibitor treatment. Furthermore, elucidating the relationship between metabolic and CSC stemness may provide a method to effectively target this critical tumor cell population.

Here, we compare the metabolic differences between LCSCs and the hepatocellular carcinoma cell line HCCLM3, and examine how metabolic/oxidative stress modulates the stemness of LCSCs.

## 2. Results

### 2.1. Enriched Sphere-Forming Cells Exhibit Stemness Properties

**Figure 1:**
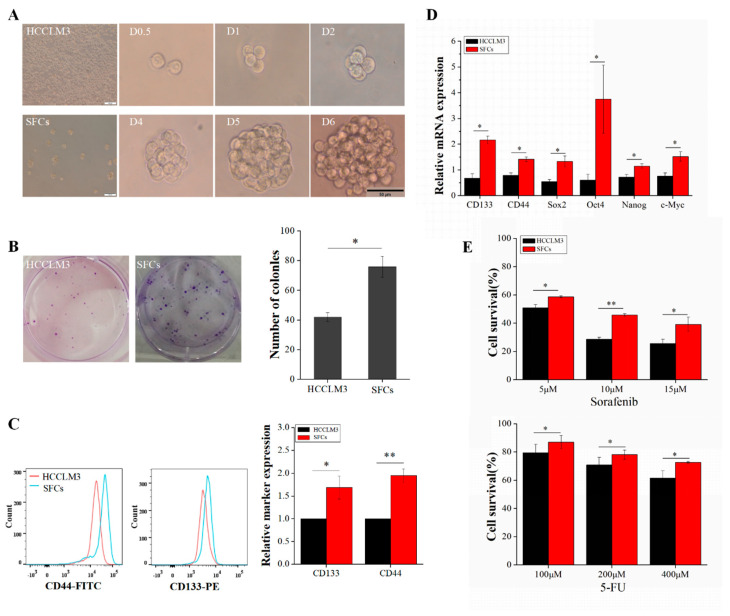
Enrichment and characterization of liver cancer stem cells (LCSCs) from HCCLM3 cells. (**A**) Morphological characteristics of HCCLM3 and sphere-forming cells (SFCs); process of sphere formation from a single P3 SFC (scale bar = 50 μm). (**B**) Comparison of the clonogenicity of HCCLM3 cells and SFCs in vitro. (**C**) Detection of the surface markers CD133 and CD44. (**D**) Detection of stemness genes. (**E**) Drug resistance analysis of SFCs and HCCLM3 cells. HCCLM3 cells and SFCs were treated with 5-FU and sorafenib for 12 h. Cell survival was determined by a CCK8 assay. Relative values are presented as the means ± standard deviation (SD) of three independent experiments. *n* = 3; * *p* < 0.05 and ** *p* < 0.01.

Within tumor tissues or tumor cells, the proportion of CSCs is quite low, generally only 0.01~2%. The further study of CSCs requires us to obtain “purified” CSCs without the loss of stem cell characteristics during continuous culture. Specifically, in this study, we employed DMEM/F12 medium to continuously generate sphere-forming cells (SFCs) from HCCLM3 cells and identify SFCs with stemness properties. SFCs were inoculated in 96-well plates at a density of 1 cell/well, and tumor subspheres were evident after 6 days. Figure 1A shows the sphere formation process of a single SFC. P3 SFCs and HCCLM3 cells efficiently formed colonies after 7 days of growth in six-well plates precoated with Matrigel (Figure 1B). Both types of cells were viable and adherent, but the colony numbers differed. The proliferation of P3 SFCs was significantly faster than that of HCCLM3 cells, and P3 SFCs exhibited enhanced colony formation. The numbers of colonies formed by 100 seeded HCCLM3 cells and SFCs were 42 ± 3 and 76 ± 7, respectively (*p* < 0.05) (Figure 1B). These results strongly indicate that P3 SFCs display capacities for self-renewal and proliferation.

Some studies suggest that CSCs are derived from mutations in adult stem/progenitor cells from the corresponding organ, as they exhibit a high expression of stemness-related genes and specific markers for adult stem/progenitor cells [11]. Therefore, the LCSC surface markers CD133 and CD44 were examined by flow cytometry. CD133 and CD44 were significantly enriched in SFCs compared with HCCLM3 cells (Figure 1C). The qRT-PCR results showed that the expression levels of the stemness-related genes Sox2, Nanog, c-Myc, and Oct4 (Figure 1D) were higher in SFCs than in HCCLM3 cells, implying that SFCs have CSCs properties.

Compared with non-stem cancer cells, CSCs exhibit an increased resistance to chemotherapy. To examine whether the chemosensitivity of HCCLM3 and SFCs differed, we performed a CCK8 assay by treating 1 × 10^4^ cells from each group with different concentrations of 5-FU and sorafenib in 96-well plates precoated with Matrigel. After a 12-h treatment with 5-FU (100, 200, and 400 µM), the survival rate of SFCs was significantly higher (1.1-fold, 1.1-fold, and 1.2-fold, respectively) than that of the corresponding HCCLM3 cells (Figure 1E). Moreover, SFCs treated with sorafenib (5, 10, and 15 µM) exhibited significantly higher survival rates (1.2-fold, 1.6-fold, and 1.6-fold, respectively) than the corresponding HCCLM3 cells (Figure 1E). The influence of SFCs on chemoresistance can be understood based on the above results and possibly explains the failure of clinical treatment to eradicate progenitors and prevent tumor regeneration.

### 2.2. Liver Cancer Stem Cells Exhibit More Robust Glycolysis than Non-Stemness Cells

**Figure 2:**
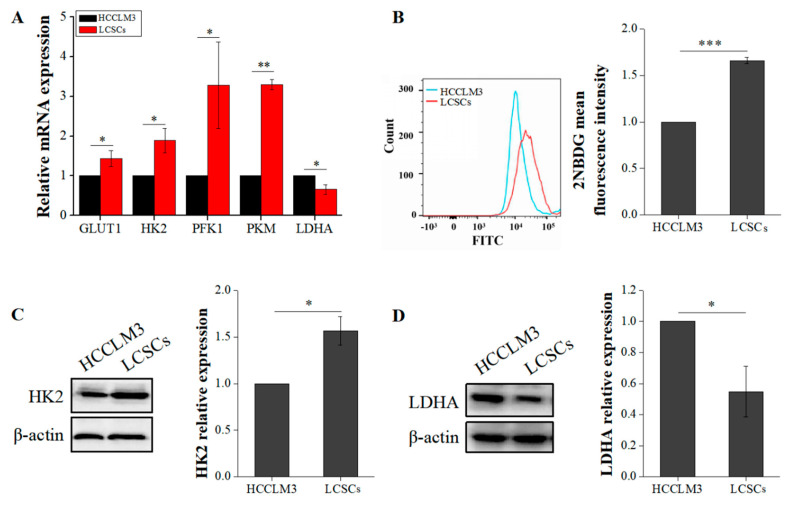
Comparison of glycolysis between HCCLM3 and LCSCs. (**A**) Comparison of glycolytic genes. (**B**) Comparison of 2-deoxy-2-[(7-nitro-2,1,3-benzoxadiazol-4-yl) amino]-D-glucose (2-NBDG) uptake. (**C**) Comparison of the glycolytic protein hexokinase 2 (HK2). (**D**) Comparison of the glycolytic protein lactate dehydrogenase A (LDHA). The values are presented as the means ± SD of three independent experiments. *n* = 3; * *p* < 0.05; ** *p* < 0.01; and *** *p* < 0.001.

Glycolysis and OXPHOS are the main pathways of energy metabolism in cells. Previous studies have shown that energy metabolism plays a crucial role in stem cell stemness maintenance [12]. However, studies on CSC energy metabolism are lacking, and this process is poorly understood. In this study, we first compared the differences in glycolysis between LCSCs and HCCLM3 cells. Hexokinase 2 (HK2), phosphofructokinase (PFK1), and pyruvate kinase (PKM), which are common indicators for detecting glycolysis, catalyze irreversible reactions in glycolysis. We compared the expression of glucose transporter 1 (GLUT1), HK2, PFK1, PKM, and lactate dehydrogenase A (LDHA) by qRT-PCR and found that the expression of these glycolytic proteins was significantly higher in LCSCs than in HCCLM3 cells (Figure 2A). Moreover, the glucose uptake capacity of LCSCs was 1.66-fold higher than that of HCCLM3 cells (*p* < 0.001) (Figure 2B). Given that HK2 is a rate-limiting enzyme in glycolysis, we also measured its protein levels by western blotting. The expression of HK2 in LCSCs was 1.63-fold higher than that in HCCLM3 cells (Figure 2C). However, the results of both qRT-PCR and western blot analyses showed that LDHA expression was significantly downregulated in LCSCs (Figure 2A,D). LDHA functions in cells to convert pyruvate produced by glycolysis into lactic acid, which is transported from the cell to the microenvironment by monocarboxylate transporters on the cell membrane. Based on the above results, we speculated that this downregulation may occur because the amount of pyruvate entering mitochondria for OXPHOS is increased. Therefore, we compared the differences between OXPHOS in HCCLM3 cells and LCSCs.

### 2.3. Liver Cancer Stem Cells Exhibit More Robust Oxidative Phosphorylation than Non-Stemness Cells

**Figure 3:**
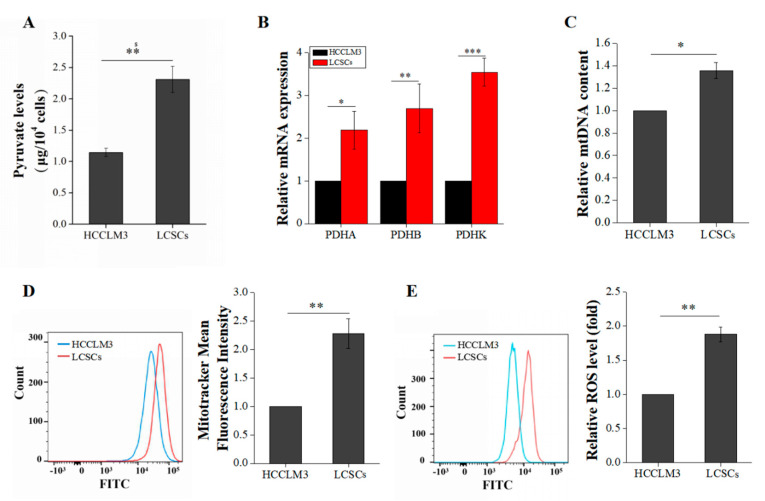
Comparison of oxidative phosphorylation (OXPHOS) between HCCLM3 and LCSCs. (**A**) Comparison of pyruvate levels. (**B**) Comparison of the pyruvate dehydrogenase complex (PDHC) expression. (**C**) Measurement of the mtDNA copy number. (**D**) Measurement of the mitochondrial mass. (**E**) Measurement of the reactive oxygen species (ROS) level. The values are presented as the means ± SD of three independent experiments. *n* = 3; * *p* < 0.05; ** *p* < 0.01; and *** *p* < 0.001.

Firstly, we compared the content of pyruvate in HCCLM3 and LCSCs. The pyruvate levels in LCSCs were significantly higher than in HCCLM3 cells (Figure 3A). The pyruvate dehydrogenase complex (PDHC) is composed of three subunits (PDHA, PDHB, and PDHK) and catalyzes the overall conversion of pyruvate to acetyl coenzyme A (acetyl-CoA), which establishes the basic connection between glycolysis and the tricarboxylic acid (TCA) cycle. A qRT-PCR was used to compare the expression levels of PDHA, PDHB, and PDHK. PDH expression was generally upregulated in LCSCs (Figure 3B). Moreover, mitochondria are organelles that exhibit very active division and fusion, and mitochondrial biogenesis is thought to be the basis for mitochondrial division and cellular energy metabolism under stress [13]. Reactive oxygen species (ROS) are a byproduct of OXPHOS metabolism in cells. Therefore, via qRT-PCR and flow cytometry, we compared the mtDNA copy number, mitochondrial contents, and ROS levels in these two types of cells. The mtDNA copy number and mitochondrial content were significantly higher (2.2-fold) in LCSCs than in HCCLM3 cells (Figure 3C,D). In addition, the ROS level was also higher (1.9-fold) in LCSCs (Figure 3E). These results suggest that our hypothesis is reasonable; LCSCs exhibit more robust glycolysis than HCCLM3 cells, thus producing more pyruvate, which is converted into acetyl-CoA A by the PDH complex and enters mitochondria for OXPHOS.

### 2.4. 2-DG Increases Liver Cancer Stem Cell Stemness by Upregulating Oxidative Phosphorylation

As the study of tumor tissue heterogeneity has become increasingly intensive, the precise targeting of CSCs is attracting interest and attention in tumor therapy. Given the critical role of the energy metabolism phenotype in maintaining stemness properties, regulating CSC energy metabolism is considered a breakthrough in targeting CSCs.

**Figure 4:**
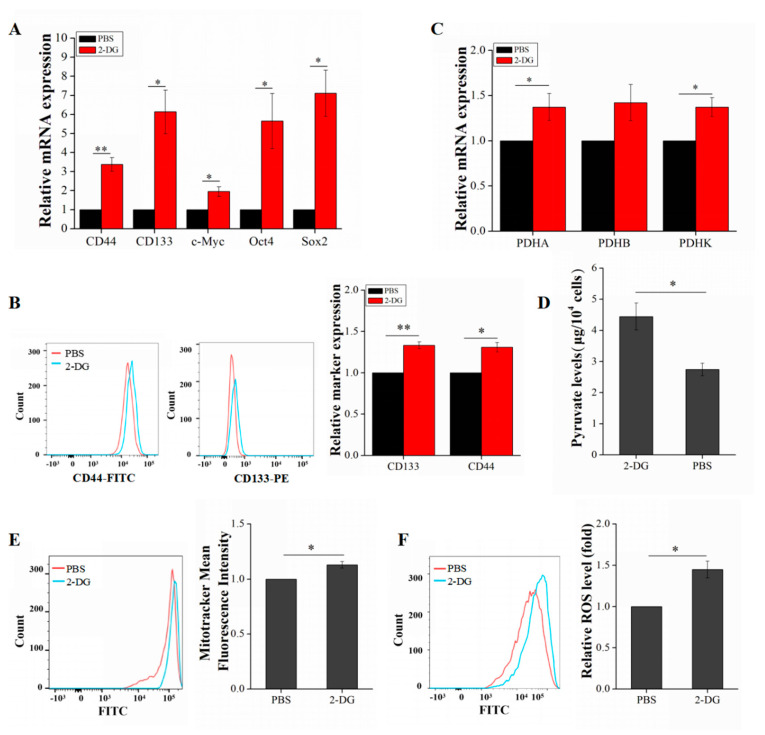
Assessment of LCSC metabolism and stemness gene expression after 2-DG treatment. (**A**) Detection of stemness genes after 2-DG treatment. (**B**) Detection of the surface markers CD133 and CD44 after 2-DG treatment. (**C**) Comparison of PDHC expression after 2-DG treatment. (**D**) Comparison of pyruvate levels after 2-DG treatment. (**E**) Measurement of the mitochondrial mass after 2-DG treatment. (**F**) Measurement of the ROS level after 2-DG treatment. The values are presented as the means ± SD of three independent experiments. *n* = 3; * *p* < 0.05; ** *p* < 0.001.

LCSCs were treated with 2-DG in order to examine the changes in energy metabolism and stemness properties before and after its use. P3 LCSCs were divided into two groups: one was treated with 2-DG and the other was treated with PBS. After treatment, the medium in both groups was replaced with complete medium, and culture was continued for 20 h. Interestingly, the qRT-PCR results implied that the expression of stemness genes was upregulated in LCSCs after 2-DG treatment (Figure 4A). The flow cytometric results demonstrated that the expression of the surface markers CD133 and CD44 was also significantly enhanced (1.33-fold and 1.31-fold, respectively) (Figure 4B). We speculated that 2-DG may inhibit glycolysis in LCSCs and induce a reliance on mitochondrial OXPHOS for energy supply and stemness maintenance. Consistent with our findings, a publication showed that 2-DG can increase OXPHOS and ROS levels in lung cancer cells [14,15,16]. To provide support for this hypothesis, we examined and compared the changes in OXPHOS after treatment with 2-DG. Both the PDHC expression and pyruvate levels were upregulated in LCSCs (Figure 4C,D). Moreover, the mitochondrial content and level of ROS generated by OXPHOS in mitochondria were increased (Figure 4E,F).

### 2.5. Mdivi-1 Reduces Liver Cancer Stem Cells Stemness by Inhibiting Oxidative Phosphorylation

To further explain the relationship between OXPHOS and the stemness of LCSCs, we treated LCSCs with Mdivi-1 and compared the changes in stemness properties. Mdivi-1 is a commonly used inhibitor of mitochondrial division and a selective inhibitor of dynamin-related protein 1 (Drp1), which is a key protein regulating mitochondrial division.

**Figure 5:**
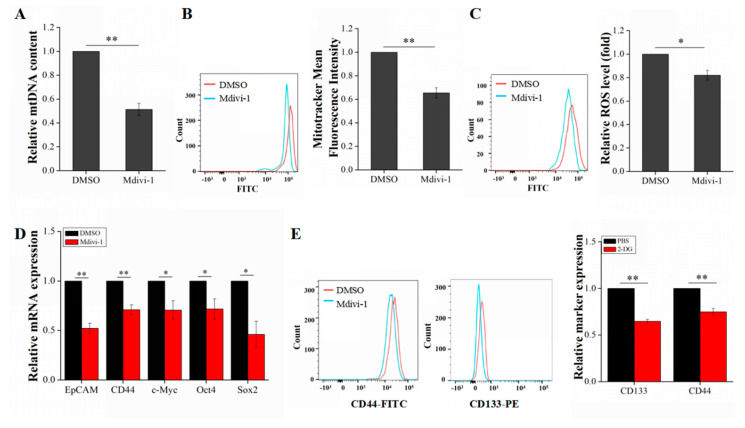
Assessment of mitochondrial biogenesis and stemness gene expression in LCSCs after Mdivi-1 treatment. (**A**) Measurement of the mtDNA copy number after Mdivi-1 treatment. (**B**) Measurement of the mitochondrial mass after Mdivi-1 treatment. (**C**) Measurement of the ROS level after Mdivi-1 treatment. (**D**) Detection of stemness genes after Mdivi-1 treatment. (**E**) Detection of the surface markers CD133 and CD44 after Mdivi-1 treatment. The values are presented as the means ± SD of three independent experiments. *n* = 4; * *p* < 0.05; and ** *p* < 0.01.

As evaluated by qRT-PCR and flow cytometry, the inhibition efficiency of Mdivi-1 was 48% and 35%, respectively (*p* < 0.01) (Figure 5A,B). These data indicated that mitochondrial division is effectively suppressed in LCSCs after treatment with Mdivi-1. The level of ROS generated by OXPHOS was also significantly reduced (*p* < 0.01) (Figure 5C). These results implied that OXPHOS was significantly inhibited in LCSCs after treatment with Mdivi-1. Furthermore, the stemness properties of LCSCs were examined. The expression of LCSC stemness genes was significantly downregulated after treatment with Mdivi-1, and the expression of the surface markers CD133 and CD44 in CSCs was significantly reduced (Figure 5D,E).

## 3. Discussion

Phenotypic and functional heterogeneity is one important feature of cancer cells and is responsible for treatment failure. CSCs are considered to be the root cause of tumor heterogeneity, because of their ability to generate the full repertoire of cancer cell types [17]. However, numerous studies have found that identifying and targeting CSCs is not simple and that permanently curing tumors relies on the complete elimination of CSCs. The separation of CSCs from tumor tissues or tumor cells is a prerequisite for and the basis of research. Therefore, in this study, we generated SFCs from HCCLM3 cells and intensely analyzed their CSC characteristics from multiple aspects. The SFCs obtained by clonal culture in vitro had superior stemness properties and conformed to the characteristics of LCSCs, unlike the HCCLM3 subpopulation.

Regulating the energy metabolism phenotype of stem cells can induce their differentiation. However, few studies have directly explored the energy metabolism phenotypes of CSCs. We conducted a careful comparison of energy metabolism phenotypes between hepatoma cells and LCSCs, focusing on glycolysis and OXPHOS. Our study suggests that LCSCs exhibit more robust glucose metabolism than non-stem hepatoma cells and that more pyruvate produced by glycolysis is converted into acetyl-CoA through PDHC and enters mitochondria for OXPHOS. Accumulating evidence indicates that glucose is an essential nutrient for CSCs, that the presence of glucose in the microenvironment can significantly increase the number of CSCs in tumor tissue, and that a lack of glucose leads to CSC death in in vitro culture [18]. The major source of intracellular ATP is mitochondrial OXPHOS. Studies have revealed that CSCs with high metastatic potential are often accompanied by more robust OXPHOS metabolism and higher ATP levels, which benefits their detachment from the basement membrane in tumor tissue to form metastatic tumors [19]. Based on findings from previous studies, we speculate that the increased levels of OXPHOS in LCSCs may be associated with their increased malignancy. Similarly, studies have reported a superior contribution of mitochondria to the maintenance of CSC stemness properties and metastatic potential and to the increased resistance of CSCs to DNA damage [20]. In addition, other publications have demonstrated that PGC1α expression is significantly upregulated in circulating tumor cells [21] and that the inhibition of PGC1α expression notably weakens the stemness properties of breast CSCs [22]. In addition to being a major source of ATP, mitochondria participate in the regulation of multiple intracellular signaling pathways, including the regulation of apoptosis through the release of cytochrome C and the release of ROS and other metabolites, such as acetyl-CoA, in order to regulate the acetylation of other proteins [23]. The increased mitochondrial content in CSCs compared with non-stem tumor cells is often accompanied by an enhanced resistance and superb self-renewal properties [24,25]. Research has indicated that, during chemotherapy, cells with more active energy metabolism in tumor tissues were more likely to survive than those with less active energy metabolism, which was the main cause of the poor patient prognosis [26]. Collectively, the above studies show that enhanced mitochondrial oxidative metabolism is essential to maintaining the biological behavior of CSCs, especially their drug resistance, metastatic potential, and stemness properties.

Furthermore, we elucidated the relationship between energy metabolism and LCSC stemness via two inhibitors of energy metabolism. Our results suggest that OXPHOS plays a crucial role in the maintenance of LCSC stemness and that 2-DG enhances LCSC stemness by upregulating OXPHOS. In contrast, Mdivi-1 reduced the levels of OXPHOS and weakened stemness by inhibiting mitochondrial fission. Our discoveries clarify the understanding that OXPHOS plays a crucial role in LCSC stemness. With the development of metabolomics, the relationship between energy metabolism and cell fate has been revealed. Specifically, the unique metabolic phenomena in tumor cells play an important role in their biological behaviors, such as proliferation, invasion, and drug resistance [27]; thus, the glycolytic inhibitor 2-DG is considered a potential and promising treatment [28,29]. Since 1950, extensive and in-depth studies on 2-DG at both basic and clinical levels have been carried out, revealing its wide-ranging effects on metabolic regulation. In addition to inhibiting glycolysis, 2-DG effectively increases the level of oxidative stress in cells, interferes with glycosylation, and induces autophagy [30,31]. Despite these findings, 2-DG is only clinically used as a chemotherapeutic adjunct, since the application of this drug as a single agent may result in drug resistance in tumor cells. Therefore, we speculate that increasing the oxidative stress levels in cells by treatment with 2-DG promotes the chemosensitivity of common tumor cells in tumor tissues and that the enhancement of CSC stemness may be the main reason for the weak clinical effects of 2-DG.

Interestingly, some publications have revealed that the key first step by which 2-DG inhibits glycolysis may result in the dual inhibition of cellular glycolysis and OXPHOS, leading to a decrease in ATP production, the prevention of cell cycle progression, and even cell death [32,33]. This view contradicts our results for two possible reasons. First, in tumor cells, the energy deficiency caused by 2-DG is insufficient to cause apoptosis, whereas it can play a role in protecting tumor cells from apoptosis because of the higher ATP levels present during apoptosis [34]. In addition, 2-DG treatment does not directly affect OXPHOS in cells. Under normoxic conditions, cells can synthesize ATP from other carbon sources, such as fatty acids or amino acids [35]. Specifically, fatty acids can be decomposed into acetyl-CoA A by β-oxidation, and acetyl-CoA thus enters mitochondria for oxidative metabolism; alternatively, the deamination or transamination of amino acids can yield acetyl-CoA or α-ketonic acid, and acetyl-CoA can then enter mitochondria for complete oxidation. Considering these previous studies, we speculate that, after treatment with 2-DG, the increased levels of oxidative stress in LCSCs are likely associated with lipid metabolism or amino acid metabolism compensation.

In this study, we show that LCSCs generated from the hepatocellular carcinoma cell line HCCLM3 and the parental cells have different metabolic characteristics, and LCSCs display markedly different sensitivities to inhibitors of glycolysis and OXPHOS metabolism. OXPHOS in mitochondria plays a crucial role in the maintenance of LCSC stemness. Treatment with 2-DG enhances the oxidative stress level in LCSCs and contributes to the promotion of stemness properties. In contrast, the mitochondrial fission inhibitor Mdivi-1 effectively induces differentiation and weakens LCSC stemness properties by reducing the level of OXPHOS. Our results provide theoretical guidance for LCSC-targeted interventions in the clinical treatment of liver cancer. However, there are limitations in this study that should be noted. Our findings in this study are from the single cell line HCCLM3. Previous studies reported that there was a significant difference in LCSC proportions among the different metastatic potential hepatocellular carcinoma cell lines [36,37,38]. Whether these LCSCs generated from different hepatocellular carcinoma cell lines exhibit different energy metabolic characteristics remains unclear. Therefore, a second cell line and/or additional hepatocellular carcinoma cell lines, which consist of different amounts of LCSCs, should be employed to solidify the generalizability and validity of our findings in this study in the future.

## 4. Materials and Methods

### 4.1. Cell Culture and Enrichment

The malignant human hepatoma cell line HCCLM3 was obtained from the Liver Cancer Institute, Zhongshan Hospital, Fudan University (Shanghai, China) and cultured in DMEM/H (Gibco, Carlsbad, CA, USA) supplemented with 10% fetal bovine serum (FBS; HyClone, Logan, UT, USA), streptomycin (100 U/mL), and penicillin (100 U/mL) in an incubator at 37 °C with 5% CO_2_. HCCLM3 cells were diluted to 1000 cells/mL in DMEM/F12 medium (Gibco, Carlsbad, CA, USA) supplemented with 20 ng/mL bFGF (Life Technologies, Carlsbad, CA, USA), 20 ng/mL EGF (PeproTech, Rocky Hill, NJ, USA), 1% N2 (Gibco, Carlsbad, CA, USA), and 2% B27 without vitamin A (Gibco, Carlsbad, CA, USA), and 2 mL of the prepared cell suspension was seeded into six-well plates for enrichment culture.

### 4.2. Sphere Formation Assay

Since LCSCs appeared spherical, we termed them sphere-forming cells (SFCs). SFCs from the enrichment culture were harvested and dissociated into single cells. In addition, one cell per well was added to a 96-well plate in DMEM/F12 medium. Cells were cultured for an additional 5–7 days in the medium until the sphere diameter was greater than 100 µm. The wells were observed daily.

### 4.3. Colony Formation Assay

To analyze colony formation, we plated HCCLM3 cells and SFCs (100 cells/well) in a six-well plate precoated with Matrigel. We assessed the colony formation ability of the cells after incubation for 7 days at 37 °C in 5% CO_2_ by counting the number of colonies (containing >70 cells) with a microscope after crystal violet staining.

### 4.4. Flow Cytometry

We applied the following monoclonal antibodies (mAbs) for flow cytometry. A phycoerythrin (PE)-conjugated mouse IgG1 isotype control antibody (Biolegend, San Diego, CA, USA) and a fluorescein isothiocyanate (FITC)-conjugated mouse IgG1 K isotype control antibody (Biolegend, San Diego, CA, USA) were used as isotype controls. In addition, a PE-conjugated human anti-CD133 antibody and an FITC-conjugated human anti-CD44 antibody (Biolegend, San Diego, CA, USA) were utilized for surface marker analysis. Flow cytometry was performed using a BD FACS Canto II (BD, San Diego, CA, USA) flow cytometer equipped with software. Side scatter and forward scatter profiles were used to eliminate cell doublets.

Cells were harvested and dissociated into single cells. Moreover, cells were stained using a reactive oxygen species (ROS) kit (Beyotime, Shanghai, China), 2-deoxy-2-[(7-nitro-2,1,3-benzoxadiazol-4-yl) amino]-D-glucose (2-NBDG; MCE, Monmouth Junctiony, NJ, USA), or Mito-Tracker Green (Beyotime, Shanghai, China), according to the manufacturer’s guidelines and were then analyzed with a BD FACS Canto II (BD, San Diego, CA, USA) flow cytometer.

### 4.5. Chemotherapeutic Sensitivity Assay

The sensitivity of HCCLM3 cells and SFCs to chemotherapeutic drugs was measured by a Cell Counting Kit-8 (CCK8; Beyotime, Shanghai, China) assay. Specifically, cells were seeded at a density of 1 × 104 cells/well in a 96-well plate precoated with Matrigel (BD, San Diego, CA, USA). Then 5-fluorouracil (5-FU) or sorafenib was separately added to the culture medium at final concentrations of 100, 200, and 400 μM (5-FU) and 5, 10, and 15 μM (sorafenib). After 12 h of treatment, the cell viability was evaluated via a CCK8 (Beyotime, Shanghai, China) assay.

### 4.6. Quantitative Real-Time PCR

Total RNA was extracted from cells using an RNA extraction kit (Bioteke, Beijing, China), and equal amounts of RNA were reverse transcribed to cDNA using Prime Script RT Master Mix (TaKaRa, Kyoto, Japan). According to the manufacturer’s instructions, a real-time quantitative reverse transcription polymerase chain reaction (qRT-PCR) was performed using a CFX96™ real-time PCR detection system (Bio-Rad, Hercules, CA, USA) with SYBR Green Master Mix (TaKaRa). The data were analyzed using CFX software (Bio-Rad, Hercules, CA, USA). The following specific primers for several genes were used: β-actin: sense 5′ GGGAAATCGTGCGTGACATT 3′, antisense 5′ TGCCCAGGAAGGAAGGCT 3′; EpCAM: sense 5′ GAATGGCTCAAAACTTGGGAGA 3′, antisense 5′ CGTTGCACTGCTTGGCCTTAA 3′; Oct4: sense 5′ GGTATTCAGCCAAACGACCATC 3′, antisense 5′ CAGCTTCCTCCACCCACTTCT 3′; Sox2: sense 5′ GCACCGCTACGACGTGAGC 3′, antisense 5′ GCCCTGGAGTGGGAGGAAGA 3′; Nanog: sense 5′ CCTATGCCTGTGATTTGTGGG 3′, antisense 5′ TTGCCTTTGGGACTGGTGG 3′; c-Myc: sense 5′ TACATCCTGTCCGTCCAAGCA 3′, antisense 5′ CACAAGAGTTCCGTAGCTGTTCAA 3′; CD133: sense 5′ TGGAGCGTCCCTTCACCC 3′, antisense 5′ TTTCTCAAAGTATCTGGATGTAGCA 3′; CD44: sense 5′ CGGACACCATGGACAAGTTT 3′, antisense 5′ CACGTGGAATACACCTGCAA 3′; GLUT1: sense 5′ TCACTGTGCTCCTGGTTCTGTTC 3′, antisense 5′ GCTCCTCGGGTGTCTTGTCA 3′; HK2: sense 5′ GCCCGCCAGAAGACATTAGA 3′, antisense 5′ CCTTGCTCAGACCTCGCTCC 3′; PFK1: sense 5′ GGAGGAACACCTTTGTCGCC 3′, antisense 5′ GTCAAAGGCTGATGGCGTCC 3′; PKM: sense 5′ GCTTCTGACCCCATCCTCTACC 3′, antisense 5′ CGTTATCCAGCGTGATTTTGAG 3′; LDHA: sense 5′ GATTCAGCCCGATTCCGTTAC 3′, antisense 5′ GAGTCCAATAGCCCAGGATGTG 3′; PDHA: sense 5′ AACCCCACAGACCATCTCATCA 3′, antisense 5′ CCTTTCCCTTTAGCACAACCTC 3′; PDHB: sense 5′ TGGAGAAGAAGTTGCCCAGTATG 3′, antisense 5′ ACCAGCCATAGCTGCACCTA 3′; and PDHK: sense 5′ TTGGCTGGATTTGGTTATGGT 3′, antisense 5′ CTTCAGGCGTGGTCTTGTAATG 3′.

### 4.7. Western Blot Analysis

Proteins were extracted in cell lysis buffer after cells were washed with PBS. Proteins were transferred to polyvinylidene fluoride (PVDF) membranes (Millipore, Bedford, MA, USA) after separation by 12% sodium dodecyl sulfate (SDS) polyacrylamide gel electrophoresis. Membranes were blocked with Quick Blocking Buffer (Willget Biotech, Beijing, China) for 1 h at room temperature. Immunoblotting was performed using primary antibodies specific for hexokinase 2 (HK2; Abcam, Cambridge, UK), lactate dehydrogenase A (LDHA; Abcam, Cambridge, UK), and horseradish-peroxidase (HRP)-conjugated secondary antibodies, and membranes were developed using a chemiluminescence (CL) reagent. A ChemiDoc MP imaging system (Bio-Rad) was used to visualize CL signals, and Image Lab 5.0 software (Bio-Rad) was used to analyze the signals.

### 4.8. 2-DG Treatment

The working concentration of the 2-deoxy-D-glucose (2-DG; MCE, Monmouth Junctiony, NJ, USA) solution was 10 nM. Passage 3 (P3) LCSCs were divided into two groups and treated with 2-DG or PBS for 3 h. After treatment, the medium was replaced with DMEM/F12 complete culture medium for 20 h, and cells were collected for subsequent analysis.

### 4.9. Mdivi-1 Treatment

The working concentration of the Mdivi-1 (a mitochondrial fission inhibitor; Beyotime, Shanghai, China) solution was 25 μM. P3 LCSCs were divided into two groups and treated with Mdivi-1 or DMSO for 8 h. After treatment, the cells were collected for subsequent analysis.

### 4.10. Determination of Pyruvate Levels

Cells were seeded at 50 × 10^4^ cells per well in a six-well plate. After 48 h, the pyruvate levels in the cells were determined using the Pyruvate Assay Kit (Solarbio, Beijing, China), according to the manufacturer’s instructions. The pyruvate levels were then normalized to the cell number.

### 4.11. Statistical Analysis

All statistically analyzed data are presented as the mean ± standard deviation values. One-way analysis of variance (ANOVA) was used to analyze group data, and a *t*-test was performed to estimate the significance of differences between the two groups. Specifically, a value of *p* < 0.05 was considered to indicate statistical significance.
